# *p* value variability and subgroup testing

**DOI:** 10.1007/s00394-021-02498-z

**Published:** 2021-02-13

**Authors:** Graham Horgan

**Affiliations:** Biomathematics and Statistics Scotland, Aberdeen, Scotland

**Keywords:** *p* value, Interaction, Subgroup

## Abstract

This article discusses the variability and randomness of *p* values, the most widely used currency of evidence in nutritional and health studies. One implication of this, the importance of always testing interaction terms when subgroups are examined and presented separately is also discussed.

**Supplementary Information:**

The online version contains supplementary material available at 10.1007/s00394-021-02498-z.

## Introduction

*p* values are ubiquitous in nutritional research, as they are across nearly all the health and biological sciences. In any issue of this journal all or almost all of the research papers include *p* values somewhere. With such an impressive breadth of usage, there comes the risk of abuse, and accordingly *p* values have come in for criticism recently [1–5]. This criticism has been less heard in nutritional science, and it is not the aim of this article to initiate such an attack here. Many of the issues raised elsewhere are general enough to apply to research of the sort presented in this and related journals. The intention here is to draw attention to the variability of *p* values, and the implications this has when repeating analyses in subgroups of a study.

## *p* values

We start by restating what a *p* value is. We suppose that we are studying whether an effect (or a difference or an association, etc.) of interest exists. The *p* value is the probability that, in the absence of such an effect, evidence in our data for this effect that is at least as strong as that we have observed could occur by chance. Thus it is a probability statement about the data, rather than a statement about the likelihood of the existence of the effect. If the latter is desired, then the Bayesian approach to data analysis [6] is needed.

It is also essential to remember that a *p* value does not indicate the strength or importance of an effect, only the evidence we have that it is not zero. The word “significant” has come to be universally used to refer to a finding that *p* < 0.05. When used by itself rather as the fuller expression “statistically significant”, this very easily gives a potentially misleading impression that the finding is important and relevant. This may or may not be so: we need additionally to examine the effect size to consider what is termed its “clinical significance” [7, 8]. A finding is important when it has this as well as statistical significance.

What this article aims to draw to attention is that *p* values are more random and unpredictable than might be realised. Those that are obtained at the end of a study might well have been different however much care and diligence there had been in carrying out the research and analysis.

To illustrate the variability of *p* values, we consider a simple experiment: we compare two treatments in separate groups of human volunteers or animals, which have been randomly assigned to the two groups. Many experiments are more complex than this, but the same *p* value variation would also be applicable there, or in observational studies. At the end of the experiment, we will compare the two groups with a *t* test. Even in a simple two-group experiment, we will often carry out a more sophisticated analysis, such as including covariates or transforming non-Normally distributed variables. Again, the same *p* value variability would occur in that case also.

We do not know before doing the experiment what the *p* value will be. We can ask what the expected distribution is, i.e. what *might* we get? If there is no treatment effect, the distribution is uniform between 0 and 1. So there is a 1 in 10 chance that it will be less than 0.1 and a 1 in 20 chance that it will be less than 0.05. That is the false-positive (type I error) risk that is unavoidable if conclusions are based on whether *p* values are less than 0.05 or not.

Now suppose that there is in fact a treatment effect with a standardised effect size of *D* = 1.2, i.e. the expected difference between the groups is 1.2 times the within group standard deviation. Let both groups have 12 volunteers or animals. With this sample size, a standard power calculation would return 80%[9], i.e. if *D* = 1.2 is true then we have a four out of five chance that the *p* value we will calculate at the end of the experiment will be < 0.05, and the correct conclusion, that the treatment has some effect, will be made. There is then a one in five chance that *p* > 0.05, leading to a false negative (type II error).

**Fig. 1:**
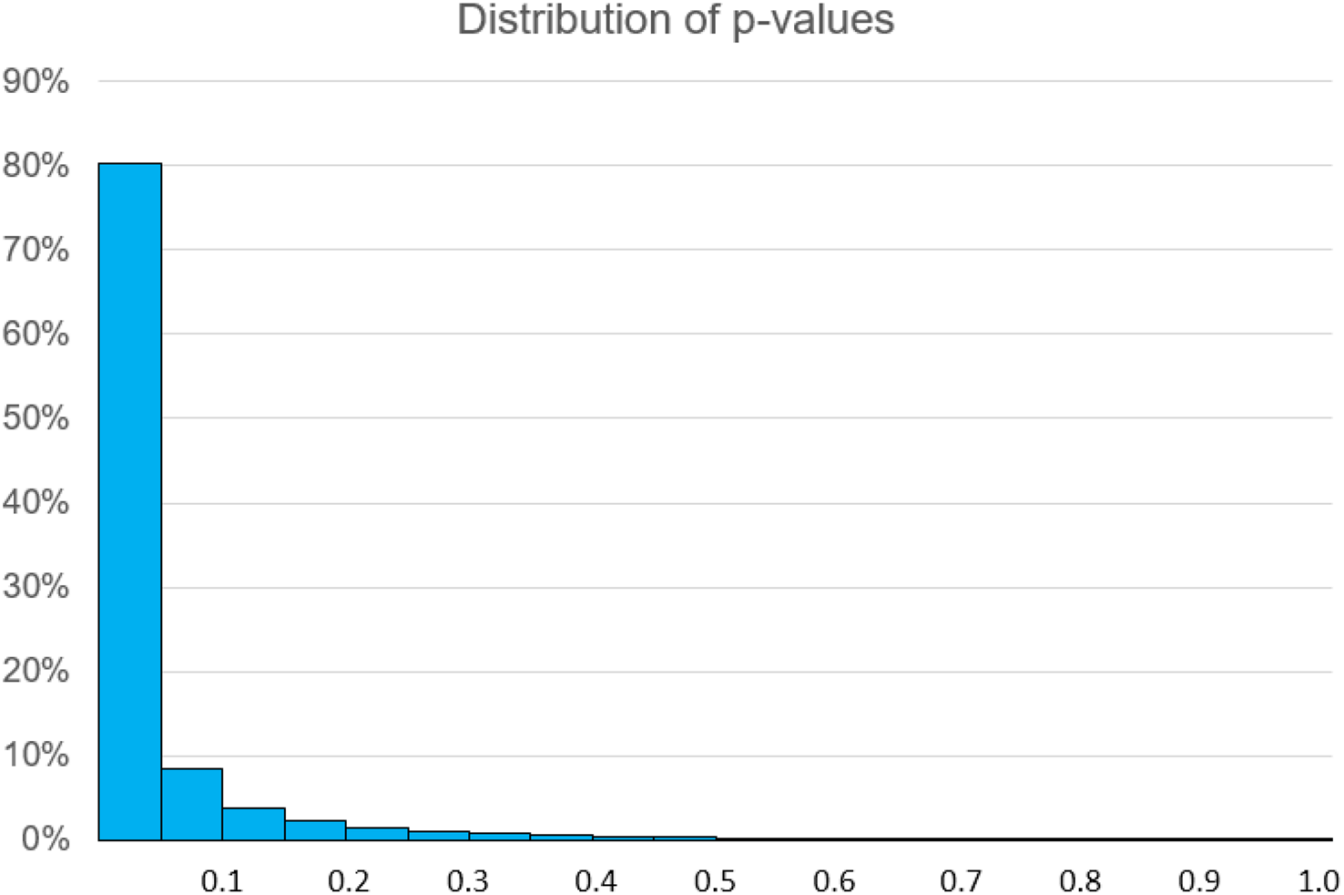
Distribution of *p* values for experiments with 80% power

Figure 1 shows the expected distribution of *p* values from this experiment. We can think of this as a histogram of the *p* values that would occur across a large number of experiments in all of which *n* = 12 per group and the power is 80%. The shape of this distribution would change only slightly for other experiments with 80% power but different sample sizes, or for different designs or different analyses. As expected, there is 80% probability that *p* < 0.05, However there is also an 8% probability that *p* > 0.2. One in five experiments studying real treatment effects with 80% power for the primary outcome will wrongly conclude that the treatment has no effect, and it will not necessarily be the case for all of these that *p* is close to 5%.

## Subgroup comparisons

One situation where we particularly need to be wary of the variability of *p* values is when we compare them in different experiments, different outcomes or different subgroups of the same experiment. It is this latter situation we examine here. Suppose that our volunteers or animals are a 50:50 mix of male and female. We may then wonder whether the treatment effect differs between them, and carry out the *t* test or other analysis separately for male and female subgroups.

The two sample sizes here will be half the total sample size and the original 80% power will be reduced to about 50% in each case. Although we are assuming here that the effect is in fact the same for both subgroups, it could readily occur that we find a significant difference in one subgroup but not in the other. Indeed there is a 50% chance that this will occur, potentially leading to erroneous conclusions. This follows from the 50% power, so the four possibilities of significance for F only, M only, neither or both are equally likely.

**Fig. 2:**
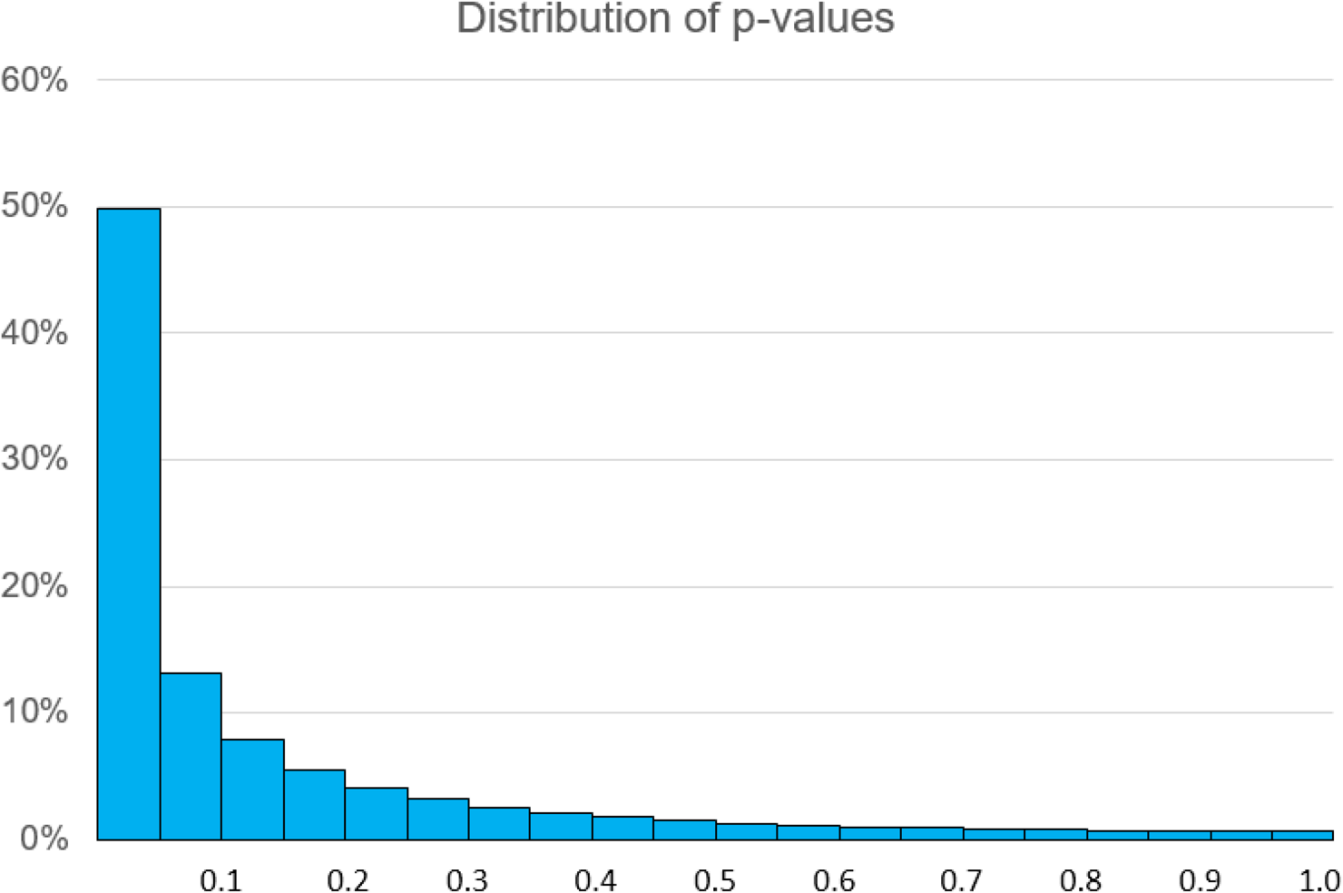
Distribution of *p* values in subgroup comparisons where power is 50%

Figure 2 shows the distribution of subgroup *p* values for a situation like this, where there is a treatment effect of *D* = 0.74, for both F and M subgroups, and 30 volunteers or animals in total per treatment group, again giving a power of 80%. The distribution shown is for tests in subgroups of size *n* = 15. It can occur quite readily that a clearly significant *p* value is seen in one group (there is about a one in four chance that it will be < 0.01) and a clearly non-significant *p* value seen in the other (with also about a one in four chance that *p* > 0.2). If this were translated into a conclusion that a treatment effect exists in one subgroup but not in the other, it would be quite wrong.

To mitigate the risk of this misleading presentation of experimental results, two strategies can be used. The first is to note that it is usually inadvisable to examine subgroups when a test of the effect in the total sample shows no significant treatment effect. Unless planned in advance and with some scientific expectation or plausibility that effects might vary between subgroups, such testing gives an appearance of what is derided as data dredging [10] and so should be done in moderation and with caution [11–13]. It can be defended as data exploration, but if so the purpose should be hypothesis generation rather than testing, and should be used to suggest further research rather than draw conclusions. The second strategy is used when subgroups have been considered in advance. In this case, we should always include a test of an interaction between the treatment factor and the subgroup factor, and proceed to subgroup comparisons only if this interaction term is significant [14]. If it is not, then this interaction term test has provided no support to any idea that the subgroups differ in their treatment response. Whenever such differences are reported without an interaction term test being presented as well, they should be viewed with caution.

## Conclusion

To conclude, this article is not calling for *p* values to be abandoned, although such a view can be heard in scientific discussion. They remain a useful currency for discussion of the evidence that scientific studies are intended to produce. However, consideration of whether they lie on one side or other of the 0.05 (or any other) threshold can be given too much emphasis, and their intrinsic variability should be remembered when drawing conclusions and making decisions, with the estimated size of any effect also being given due attention.

## Supplementary Information

Below is the link to the electronic supplementary material.

Supplemental material: A spreadsheet which calculates p-value distributions for simple two group comparisons, as in the figures, is available from the journal website. (XLSM 105 KB)
